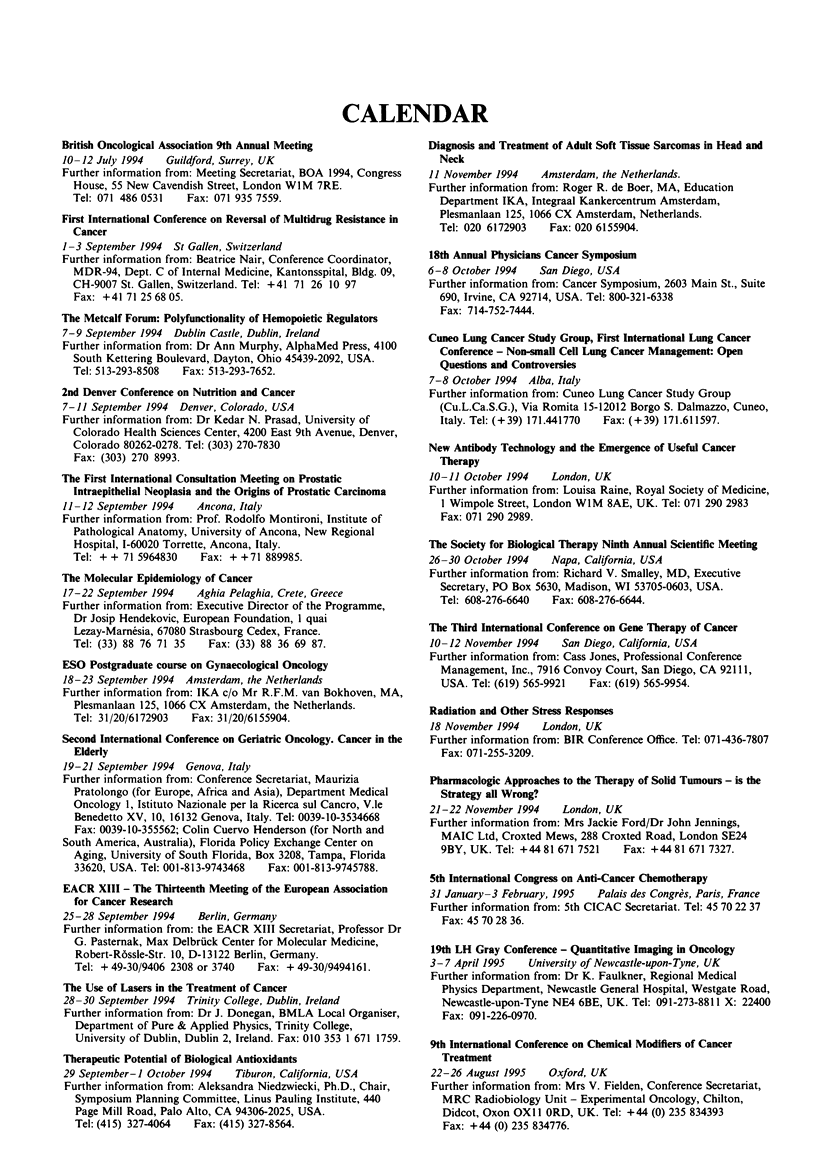# Calendar

**Published:** 1994-07

**Authors:** 


### CALENDAR

**British Oncological Association 9th Annual Meeting**

*10–12 July 1994 Guildford, Surrey, UK*

Further information from: Meeting Secretariat, BOA 1994, Congress House, 55 New Cavendish Street, London W1M 7RE. Tel: 071 486 0531 Fax: 071 935 7559.

**First International Conference on Reversal of Multidrug Resistance in Cancer**
*1–3 September 1994 St Gallen, Switzerland*
Further information from: Beatrice Nair, Conference Coordinator, MDR-94, Dept. C of Internal Medicine, Kantonsspital, Bldg. 09, CH-9007 St. Gallen, Switzerland. Tel: +41 71 26 10 97 Fax: +41 71 25 68 05.

**The Metcalf Forum: Polyfunctionality of Hemopoietic Regulators**

*7–9 September 1994 Dublin Castle, Dublin, Ireland*

Further information from: Dr Ann Murphy, AlphaMed Press, 4100 South Kettering Boulevard, Dayton, Ohio 45439-2092, USA. Tel: 513-293-8508 Fax: 513-293-7652.

**2nd Denver Conference on Nutrition and Cancer**
*7–11 September 1994 Denver, Colorado, USA*
Further information from: Dr Kedar N. Prasad, University of Colorado Health Sciences Center, 4200 East 9th Avenue, Denver, Colorado 80262-0278. Tel: (303) 270-7830 Fax: (303) 270 8993.

**The First International Consultation Meeting on Prostatic Intraepithelial Neoplasia and the Origins of Prostatic Carcinoma**

*11–12 September 1994 Ancona, Italy*

Further information from: Prof. Rodolfo Montironi, Institute of Pathological Anatomy, University of Ancona, New Regional Hospital, I-60020 Torrette, Ancona, Italy. Tel: + + 71 5964830 Fax: + +71 889985.

**The Molecular Epidemiology of Cancer**
*17–22 September 1994 Aghia Pelaghia, Crete, Greece*
Further information from: Executive Director of the Programme, Dr Josip Hendekovic, European Foundation, 1 quai Lezay-Marnésia, 67080 Strasbourg Cedex, France. Tel: (33) 88 76 71 35 Fax: (33) 88 36 69 87.

**ESO Postgraduate course on Gynaecological Oncology**
*18–23 September 1994 Amsterdam, the Netherlands*
Further information from: IKA c/o Mr R.F.M. van Bokhoven, MA, Plesmanlaan 125, 1066 CX Amsterdam, the Netherlands. Tel: 31/20/6172903 Fax: 31/20/6155904.

**Second International Conference on Geriatric Oncology. Cancer in the Elderly**
*19–21 September 1994 Genova, Italy*
Further information from: Conference Secretariat, Maurizia Pratolongo (for Europe, Africa and Asia), Department Medical Oncology 1, Istituto Nazionale per la Ricerca sul Cancro, V.le Benedetto XV, 10, 16132 Genova, Italy. Tel: 0039-10-3534668 Fax: 0039-10-355562; Colin Cuervo Henderson (for North and South America, Australia), Florida Policy Exchange Center on Aging, University of South Florida, Box 3208, Tampa, Florida 33620, USA. Tel: 001-813-9743468 Fax: 001-813-9745788.

**EACR XIII – The Thirteenth Meeting of the European Association for Cancer Research**

*25–28 September 1994 Berlin, Germany*

Further information from: the EACR XIII Secretariat, Professor Dr G. Pasternak, Max Delbrück Center for Molecular Medicine, Robert-Rössle-Str. 10, D-13122 Berlin, Germany. Tel: + 49-30/9406 2308 or 3740 Fax: + 49-30/9494161.

**The Use of Lasers in the Treatment of Cancer**
*28–30 September 1994 Trinity College, Dublin, Ireland*
Further information from: Dr J. Donegan, BMLA Local Organiser, Department of Pure & Applied Physics, Trinity College, University of Dublin, Dublin 2, Ireland. Fax: 010 353 1 671 1759.

**Therapeutic Potential of Biological Antioxidants**
*29 September–1 October 1994 Tiburon, California, USA*
Further information from: Aleksandra Niedzwiecki, Ph.D., Chair, Symposium Planning Committee, Linus Pauling Institute, 440 Page Mill Road, Palo Alto, CA 94306-2025, USA. Tel: (415) 327-4064 Fax: (415) 327-8564.

**Diagnosis and Treatment of Adult Soft Tissue Sarcomas in Head and Neck**
*11 November 1994 Amsterdam, the Netherlands.*
Further information from: Roger R. de Boer, MA, Education Department IKA, Integraal Kankercentrum Amsterdam, Plesmanlaan 125, 1066 CX Amsterdam, Netherlands. Tel: 020 6172903 Fax: 020 6155904.

**18th Annual Physicians Cancer Symposium**
*6–8 October 1994 San Diego, USA*
Further information from: Cancer Symposium, 2603 Main St., Suite 690, Irvine, CA 92714, USA. Tel: 800-321-6338 Fax: 714-752-7444.

**Cuneo Lung Cancer Study Group, First International Lung Cancer Conference – Non-small Cell Lung Cancer Management: Open Questions and Controversies**

*7–8 October 1994 Alba, Italy*

Further information from: Cuneo Lung Cancer Study Group (Cu.L.Ca.S.G.), Via Romita 15-12012 Borgo S. Dalmazzo, Cuneo, Italy. Tel: (+39) 171.441770 Fax: (+39) 171.611597.

**New Antibody Technology and the Emergence of Useful Cancer Therapy**

*10–11 October 1994 London, UK*

Further information from: Louisa Raine, Royal Society of Medicine, 1 Wimpole Street, London W1M 8AE, UK. Tel: 071 290 2983 Fax: 071 290 2989.

**The Society for Biological Therapy Ninth Annual Scientific Meeting**

*26–30 October 1994 Napa, California, USA*

Further information from: Richard V. Smalley, MD, Executive Secretary, PO Box 5630, Madison, WI 53705-0603, USA. Tel: 608-276-6640 Fax: 608-276-6644.

**The Third International Conference on Gene Therapy of Cancer**
*10–12 November 1994 San Diego, California, USA*
Further information from: Cass Jones, Professional Conference Management, Inc., 7916 Convoy Court, San Diego, CA 92111, USA. Tel: (619) 565-9921 Fax: (619) 565-9954.

**Radiation and Other Stress Responses**

*18 November 1994 London, UK*

Further information from: BIR Conference Office. Tel: 071-436-7807 Fax: 071-255-3209.

**Pharmacologic Approaches to the Therapy of Solid Tumours – is the Strategy all Wrong?**

*21–22 November 1994 London, UK*

Further information from: Mrs Jackie Ford/Dr John Jennings, MAIC Ltd, Croxted Mews, 288 Croxted Road, London SE24 9BY, UK. Tel: +44 81 671 7521 Fax: +44 81 671 7327.

**5th International Congress on Anti-Cancer Chemotherapy**

*31 January–3 February, 1995 Palais des Congrès, Paris, France*

Further information from: 5th CICAC Secretariat. Tel: 45 70 22 37 Fax: 45 70 28 36.

**19th LH Gray Conference – Quantitative Imaging in Oncology**
*3–7 April 1995 University of Newcastle-upon-Tyne, UK*
Further information from: Dr K. Faulkner, Regional Medical Physics Department, Newcastle General Hospital, Westgate Road, Newcastle-upon-Tyne NE4 6BE, UK. Tel: 091-273-8811 X: 22400 Fax: 091-226-0970.

**9th International Conference on Chemical Modifiers of Cancer Treatment**
*22–26 August 1995 Oxford, UK*
Further information from: Mrs V. Fielden, Conference Secretariat, MRC Radiobiology Unit – Experimental Oncology, Chilton, Didcot, Oxon OX11 0RD, UK. Tel: +44 (0) 235 834393 Fax: +44 (0) 235 834776.